# Harnessing historical data to derive reference limits – A comparison of e-norms to traditionally derived reference limits

**DOI:** 10.1016/j.cnp.2024.04.001

**Published:** 2024-04-16

**Authors:** Ø. Dunker, T.S. Szczepanski, H.O.P. Do, P. Omland, M.U. Lie, T. Sand, J.F. Jabre, K.B. Nilsen

**Affiliations:** a Department of Research and Innovation, Division of Neuroscience, Oslo University Hospital, Oslo, Norway; b Faculty of Medicine, University of Oslo, Oslo, Norway; c Department of Neurology and Clinical Neurophysiology, Oslo University Hospital, Oslo, Norway; d Department of Neuromedicine and Movement Science, Faculty of Medicine and Health Sciences, Norwegian University of Science and Technology, Trondheim, Norway; e Department of Neurology and Clinical Neurophysiology, St. Olavs Hospital, Trondheim, Norway; f Department of Radiology and Nuclear Medicine, St. Olavs Hospital, Trondheim University Hospital, Trondheim, Norway; g Formerly, Department of Neurology, David Geffen School of Medicine at UCLA, Los Angeles, CA, USA

**Keywords:** E-norms, Reference limits, Nerve conduction studies, DIGMINE

## Abstract

**Objective:**

Nerve conduction studies (NCS) require valid reference limits for meaningful interpretation. We aimed to further develop the extrapolated norms (e-norms) method for obtaining NCS reference limits from historical laboratory datasets for children and adults, and to validate it against traditionally derived reference limits.

**Methods:**

We compared reference limits obtained by applying a further developed e-norms with reference limits from healthy controls for the age strata’s 9–18, 20–44 and 45–60 years old. The control data consisted of 65 healthy children and 578 healthy adults, matched with 1294 and 5628 patients respectively. Five commonly investigated nerves were chosen: The tibial and peroneal motor nerves (amplitudes, conduction velocities, F-waves), and the sural, superficial peroneal and medial plantar sensory nerves (amplitudes, conduction velocities). The datasets were matched by hospital to ensure identical equipment and protocols. The e-norms method was adapted, and reference limit calculation using both ±2 SD (original method) and ±2.5 SD (to compensate for predicted underestimation of population SD by the e-norms method) was compared to control data using ±2 SD. Percentage agreement between e-norms and the traditional method was calculated.

**Results:**

On average, the e-norms method (mean ±2 SD) produced slightly stricter reference limits compared to the traditional method. Increasing the e-norms range to mean ±2.5 SD improved the results in children while slightly overcorrecting in the adult group. The average agreement between the two methods was 95 % (±2 SD) and 96 % (±2.5 SD).

**Conclusions:**

The e-norms method yielded slightly stricter reference limits overall than ones obtained through traditional methods; However, much of the difference can be attributed to a few outlying plots where the raters found it difficult to apply e-norms correctly. The two methods disagreed on classification of 4–5% of cases. Our e-norms software is suited to analyze large amounts of raw NCS data; it should further reduce bias and facilitate more accurate ratings.

**Significance:**

With small adaptations, the e-norms method adequately replicates traditionally derived reference limits, and is a viable method to produce reference limits from historical datasets.

## Highlights

• With small adaptations, the e-norms method adequately replicates traditionally derived reference limits.
• The e-norms method allows for reference limits to be created from historical datasets.
• Software was written to further develop the e-norms method and to ease adoption.

## 1 Introduction

Nerve conduction studies (NCS) are important for neuromuscular diagnostics worldwide. Valid reference limits are a prerequisite for correct interpretation of NCS. Reference limits are traditionally obtained from a convenience sample of healthy subjects (Dillingham et al., 2016, Dorfman and Robinson, 1997), but are only ecologically valid when the protocol, equipment and subjects are similar to the patients investigated in the clinic (Geffre et al., 2009, Horowitz et al., 2010). Thus, traditional reference limits are ideally created locally by recruiting hundreds or sometimes thousands of subjects. Needless to say, this method of developing reference limits by direct a priori sampling is impractical, time-consuming and resource-intensive. In response to the need for a better way of obtaining reference limits, different data-driven approaches have been proposed since the early 60′s (Hoffmann, 1963, Jones et al., 2019). More recently, a novel method of leveraging historical patient data was suggested (Jabre et al., 2015), which may allow each laboratory to more easily calculate their own reference limits.

In the extrapolated norms (e-norms) method, one transforms the NCS measurements from a historical dataset into an ascending cumulative density curve. This creates an inverted S-curve where abnormal values make up the tails, and a defined, flat, middle plateau represents a cluster of patients with expected normal NCS measurements. In e-norms, the plateau is identified through fitting a tangent, with visual aid from plotting the first order differences on the same graph. Other methods apply different techniques to identify the plateau, such as the extrapolated reference values method (E-Ref) (Nandedkar et al., 2018) and the multivariate extrapolated reference values methods (Nandedkar et al., 2021), but the theoretical underpinning is the same.

Several validation studies and applications of the e-norms method have been published on various types of data (e.g. (Jabre and Bland, 2021, Jabre et al., 2020, Pitt and Jabre, 2017, Punga et al., 2019, Shammas and Jabre, 2020)). However, a true test of the method’s concurrent validity and practicality for NCS calls for a large-scale validation of historical data gathered over many years, against traditionally obtained reference limits. This is made possible by the Norwegian DIGMINE project database which contains NCS records on more than 220,000 patients. Such validation efforts could quantify the real-world effects of applying e-norms reference limits, i.e., changes in proportions of positive and negative NCS findings. It could also give the opportunity to refine or revise the method in light of the results from different subgroup analyses, and to streamline the e-norms analyses of substantial amounts of laboratory data.

Thus, the aims of this study were to further refine the e-norms method, validate the method against traditionally sampled reference limits from children and adults; and to facilitate adoption by developing simple-to-use software that automates the e-norms method as much as possible.

## 2 Methods

### 2.1 Overview

We compared reference limits obtained by use of the e-norms method to traditionally calculated reference limits for commonly investigated NCS measures in the lower limbs. Two datasets from healthy controls and two historical laboratory datasets were matched from the same clinical neurophysiology laboratories in Norway. We created a semi-automated pipeline in Python for data transformation and e-norms analysis. The e-norms method was further developed by hiding the graph axes from the rater, and by adding two visual aids to more reliably identify the plateau of the curve: drawing a moving average of the first order derivatives, and by overlaying a 3rd order polynomial curve. The current study is a quality assurance study, approved by the Data Protection Officers at the two hospitals.

### 2.2 Control samples

#### 2.2.1 Children, 9–18 years

The control group for children and adolescents (n = 65) was recruited as part of a study on physical activity and fitness among childhood cancer survivors in Oslo (Andries et al., 2023). They consisted of age– and sex-matched, self-reported healthy friends or siblings of the childhood cancer survivors, and were recruited by the survivors and their family. The controls were screened by study personnel for heart–, lung–, and muscle disease, polyneuropathy and history of malignancy. NCS was performed on the right ulnar–, peroneal– and tibial motor nerves (F-waves, amplitude, conduction velocity, distal latency), and the ulnar–, radial–, superficial peroneal–, sural– and medial tibial sensory nerves (amplitude, conduction velocity).

#### 2.2.2 Adults, 20–60 years

The adult controls (n = 578) were recruited between 2012 and 2022. They were either healthy subjects, or patients referred to one of two Departments of Neurology in Mid-Norway (Ålesund or Trondheim) for non-specific symptoms without known disease (malignancy, diabetes, connective tissue disease, etc.), found to be free of any neurological diagnosis after examination. Identical procedures were used in both laboratories, and were overseen by the same senior consultant clinical neurophysiologist. NCS data was available from ulnar–, median,– peroneal– and tibial motor nerves (F-waves, amplitude, conduction velocity, distal latency), and the ulnar–, median–, radial–, sural–, peroneal– and medial tibial sensory nerves (amplitude, conduction velocity).

### 2.3 Historical dataset

The historical dataset for the e-norms analyses consisted of patients examined at the outpatient clinical neurophysiology clinics at Oslo university hospital (children) and St. Olavs hospital or Ålesund hospital (adults) in Norway between February 1997 and February 2021. It is a *mixed dataset*, consisting of both normal and abnormal NCS readings. Healthy controls present in the historical dataset were removed to avoid bias.

### 2.4 Further development of the e-norms method

**Fig. 1:**
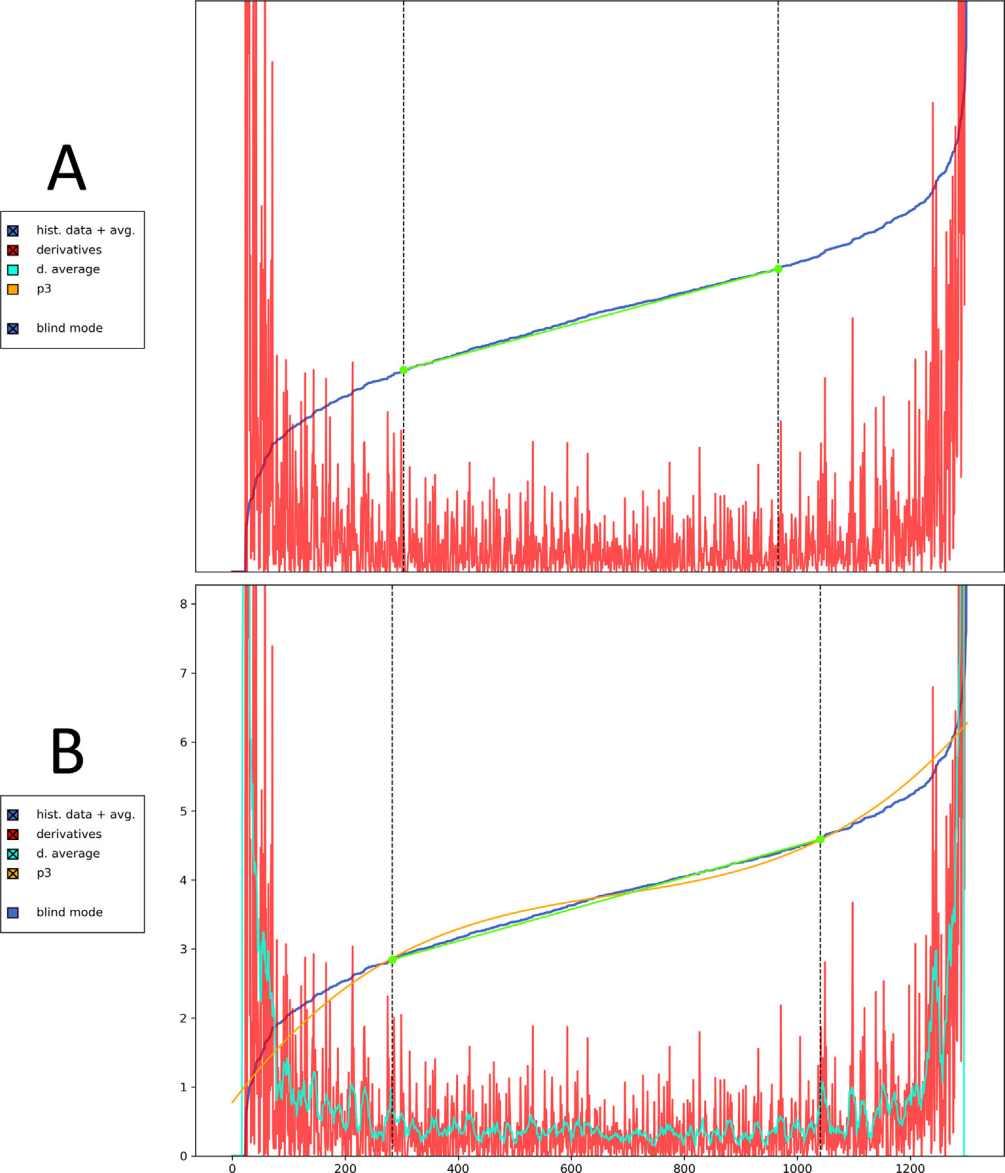
Two example instances of the e-norms method in the new dashboard. The vertical dotted lines mark the inflection point from the tangent, set by the rater. **A:** Blind e-norms rating with the S-curve, first order derivatives and a fitted tangent. **B:** E-norms with visible axis values, and with the addition of a moving average of the derivatives and a 3rd order polynomial curve (p3).

The e-norms method was first described in detail by Jabre et al. (2015). In brief: the method relies on historical data from patient examinations. The values are sorted in ascending order and plotted against rank order. This produces an inverted S-curve with steep left- and right tails, and a middle part with a steady slope (a “plateau”) that visualizes the clustering of normal measurements (Fig. 1). Next, the first-order derivatives are plotted on the same graph, by calculating the consecutive differences between the values (value2 – value1, value3 – value2 etc.). To identify the bounds of the plateau, a tangent is fitted to the plateau by eye, and matched with the interval of lowest first-order derivatives. These bounds, or points of inflection from the tangent, mark the interval where one would expect to find normal values. The values from these patients are then extracted, and traditional calculations to determine reference limits can be applied (mean ±2 SD).

We combined three adaptations to the e-norms method. First, we added the option to hide graph axes’ values, to reduce confirmation bias by the rater. With visual feedback in e.g. mV or m/s, the rater may subconsciously decide on markers that tend toward known reference limits. Furthermore, since the e-norms method relies on manual identification of the inflection points, reliability may be improved by providing the user with more technical aids. The first addition is to add a moving average to the first-order differences; Smoothing out the data with a moving average makes it easier for the user to identify when a real trend occurs. We applied a right-aligned rolling mean with a rolling window width of 0.1 % of the sample. The second addition is to overlay a 3rd order polynomial function, which will tend to intersect at the inflection points. Thus, for S-curves that are difficult to analyze (e.g. low-resolution because of low *n* or NCS latency measures), the user now has four visual aids at their disposal that can be combined to help identify the plateau.

The method itself, including data transformations, was automated to the extent possible for a method with manual determining of inflection points. The program was written in Python v3.8.5 and can be found at https://github.com/OUSAppliedNeuro/enorms.git.

### 2.5 Analyses

A balance was struck between having large enough control samples and when one can reasonably expect an age-related change in nervous function: the data was stratified into groups of 9–18, 20–44 and 45–60 years, but we did not stratify for height or sex. NCS measures from five commonly tested nerves in the lower limbs were included in the analysis: the tibial– and peroneal motor nerves (amplitudes, conduction velocities, F-min latency), and the sural, superficial peroneal and the medial plantar sensory nerves (amplitudes, conduction velocities). The control sets for the children and adults were matched against historical datasets from the same hospitals to ensure homogeneity in data collection, i.e., Oslo and Trondheim, respectively. Since most of the NCS distributions were skewed, they were described by their median with interquartile ranges.

The e-norms method was applied by two of the authors (H.O.P.D., P.O.). First, the raters met and agreed upon how to apply the e-norms method, using example graphs. They then separately applied the e-norms method with hidden axes (i.e., blinded to nerve and NCS measure, as well as each other’s ratings), but with the addition of moving averages and 3rd order polynomials when the raters deemed it useful. Afterwards, the blinded graphs were compared with regard to methodological soundness. When the e-norms plateaus were similar (visually and with < 10 % difference in plateau size), the average was reported (a final e-norms plateau was created using the average of the two top and bottom coordinates). When the graphs were clearly visually dissimilar, the differences were discussed and a third blind rater (Ø.D.) chose the plateau that was deemed to best adhere to the e-norms method. To ensure valid identification of the e-norms plateaus, only historical datasets with n > 100 were analyzed.

#### 2.5.1 Calculation of reference limits from controls

The control data was transformed to normality as necessary, with a small constant (+0.1) added to any log-transformed values to ensure proper transformation. Mean ±2 SD was calculated on the transformed data, and this reference limit was back-transformed by the opposite mathematical function. Reference limits were calculated for every NCS measure and each of the subgroups.

#### 2.5.2 Comparisons of the two methods

A mock real-world comparison of traditional– and e-norms derived reference limits was performed by calculating how many individual NCS measures would be classified as abnormal in the historical dataset, and the abnormality ratio between them (% abnormal using e-norms derived limits / % abnormal using traditional reference limits). The data was LN-transformed before calculating the abnormality ratios, and the result back-transformed by its opposite function (EXP). We did not directly compare the central tendency or variability of the distributions between the two groups: the e-norms distribution will always have low variance, and it is the final combination of mean and SD that affects diagnostics. Since SD calculated from a truncated distribution (measures classified as “healthy” by enorms) underestimates “real” SD, we also calculated mean ±2.5 SD limits to evaluate whether a simple SD-scaling would improve agreement between the two methods. Average percentage agreement was calculated between the traditionally derived reference limits and the e-norms method.

## 3 Results

Six data sets were created from control subjects and historical data (Table 1). The historical dataset of patients between 20–44 years old had fewer female patients than the control set. For those between 45 and 60 years old, the historical dataset of patients were on average older, taller and had fewer female patients than the control set.

**Table 1:** Demographics of the control subjects and historical patient data.

	**Controls**			**Historical data**		
	**Oslo**	**Trondheim**		**Oslo**	**Trondheim**	
	**9**–**18**	**20**–**44**	**45**–**60**	**9**–**18**	**20**–**44**	**45**–**60**
	**n = 65**	**n = 339**	**n = 239**	**n = 1294**	**n = 2283**	**n = 3345**
**Age, y, mean (SD)**	13.7 (2.6)	34.4 (6.6)	51.2 (4.4)	14.3 (3.0)	34.7 (6.9)	53.3 (4.6)*
**Height, cm, mean (SD)**	161.2 (14.3)	171.8 (8.5)	170.8 (8.4)	162.6 (15.1)	173.5 (9.6)	174.1 (9.2)*
**Sex, female, n (%)**	31 (48)	238 (70)	167 (70)	628 (49)	1241 (54)*	1586 (47)*

*Different from controls (p < 0.01).

The improved e-norms program launches a dashboard for performing the e-norms analysis (Fig. 1). The dashboard contains the option to toggle graph axes, add a moving average of the derivatives, and to overlay a 3rd order polynomial line.

**Fig. 2:**
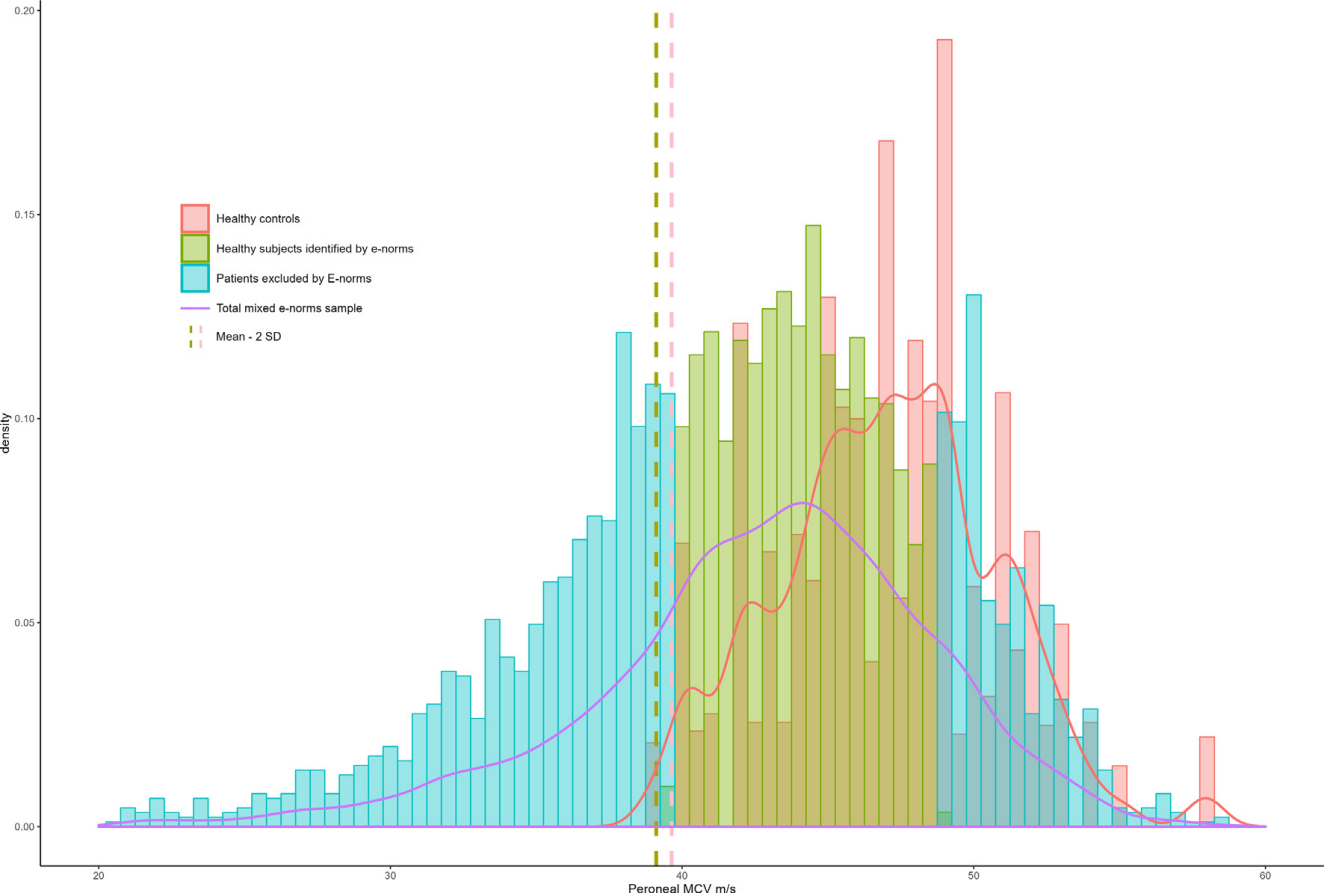
Example histograms showing the peroneal motor conduction velocity distribution of individuals aged 50–59 years. Divided into the e-norms plateau (n = 2837), those excluded by the e-norms method (n = 1755), and healthy controls (n = 97, bootstrapped to n = 2821 for easier visual comparison).

Extracting reference limits from historical data with e-norms can be visualized with histograms (Fig. 2). For the example of peroneal conduction velocity, healthy values tend to be approximately normally distributed (pink histogram), with a slight tail towards “super normality”. The distribution of the historical dataset is slightly skewed (blue and green histograms): since the values are derived from patients referred to the hospital, the distribution has a longer tail of abnormal measures. The e-norms method cuts off both tails, resulting in a truncated distribution with low variance (green histogram).

When compared to traditional reference limits, the e-norms derived limits (±2 SD) were stricter on average (Tables 2 and 3), especially for F-waves and sensory amplitudes, and for medial plantar nerve conduction velocity in children. The average percentage agreement between the traditional reference limits and the e-norms derived limits were 95 %. The average abnormality ratio between the e-norms method (±2 SD) and the traditional method for all NCS measures on average were 1.30 for children (range 0.9–3.2) and 1.14 for adults (range 0.5–3.3), i.e. 15–30 % more NCS readings classified as abnormal by the e-norms methods compared to the traditional method. The biggest differences were found for tibial and peroneal F-waves, peroneal nerve conduction velocity and medial plantar nerve conduction velocity in children, and sural and superficial peroneal sensory nerve readings in adults.

**Table 2:** Comparison of NCS reference limits in children 9–18 years’ old.

	**Traditional**					**E-norms**				
**Children 9**–**18 years**	n	Median	IQR	Reference limit (±2 SD)^a^	% abnormal in mixed dataset (±2 SD)^b^	n	Median	IQR	Reference limit (±2 SD)^a^	% abnormal in mixed dataset (±2 SD)^b^
**Motor nerves**										
**Tibial nerve**										
Amplitude, mV	63	13.5	5.2	7.5	27	1658	10.7	3.6	6.6	24
Conduction velocity, m/s	62	48.4	5.3	41.0	11	1419	48.1	4.2	43.0	16
F-min, ms	63	39.4	7.4	51.8	9	1485	42.7	5.7	48.6	14

**Peroneal nerve**										
Amplitude, mV	63	4.4	2.0	2.4	22	1683	4.1	1.5	2.2	20
Conduction velocity, m/s	63	50.9	4.8	43.2	16	1414	48.8	4.1	43.4	17
F-min, ms	62	40.0	7.4	50.1	10	1321	42.1	5.7	46.7	19

**Sensory nerves**										
**Sural nerve**										
Amplitude, µV	63	15.4	7.9	6.6	16	1301	14.3	6.5	7.3	18
Conduction velocity, m/s	63	56.0	8.5	44.7	9	1271	54.2	6.5	46.3	12

**Superficial peroneal nerve**										
Amplitude, µV	63	8.4	5.1	4.0	12	687	8.4	3.8	4.4	14
Conduction velocity, m/s	63	53.4	6.8	44.3	11	1029	50.9	5.6	44.0	10

**Medial plantar nerve**										
Amplitude, µV	63	10.1	5.9	3.8	13	228	8.4	3.7	4.3	15
Conduction velocity, m/s	63	59.6	9.2	45.3	6	222	59.6	6.4	50.9	18

IQR, inter-quartile range.

^a^Limit = Mean ±2 SD on transformed data for non-Gaussian distributions, retransformed to original scale.

^b^What percentage of NCS readings in the historical laboratory population would be classified as abnormal by the method.

**Table 3:** Comparison of NCS reference limits in adults 20–44 and 45–60 years’ old.

	**Traditional**					**E-norms**				
**Adults 20**–**44 years**	n	Median	IQR	Reference limit (±2 SD)^a^	% abnormal in mixed dataset (±2 SD)^b^	n	Median	IQR	Reference limit (±2 SD)^a^	% abnormal in mixed dataset (±2 SD)^b^
**Motor nerves**										
**Tibial nerve**										
Amplitude, mV	144	12.6	5.6	5.8	11	3169	10.6	4.0	5.9	11
Conduction velocity, m/s	142	50.0	4.4	43.2	18	3121	48.2	4.5	44.1	22
F-min, ms	144	43.8	3.8	51.2	13	3223	44.6	4.0	49.7	19

**Peroneal nerve**										
Amplitude, mV	146	5.8	2.5	2.8	16	3272	5.1	1.8	2.9	17
Conduction velocity, m/s	145	48.5	3.3	43.1	19	3072	47.9	4.4	42.0	15
F-min, ms	142	42.5	4.5	50.8	10	3051	43.3	4.5	49.0	16

**Sensory nerves**										
**Sural nerve (orthodromic)**										
Amplitude, µV	146	11.7	8.8	2.6	6	2805	7.2	3.3	3.8	13
Conduction velocity, m/s	146	53.9	7.9	42.5	8	2798	51.9	7.5	42.9	9

**Superficial peroneal nerve**										
Amplitude, µV	122	8.3	6.1	2.9	7	767	8.0	3.7	4.1	15
Conduction velocity, m/s	122	52.2	7.1	43.0	19	758	48.3	5.3	41.8	13

**Medial plantar nerve**										
Amplitude, µV	122	8.8	7.5	1.2	10	1269	4.5	3.6	1.2	9
Conduction velocity, m/s	122	56.7	8.7	45.3	14	1268	54.2	5.8	46.6	18

**Adults 45**–**60 years**										
**Motor nerves**										
**Tibial nerve**										
Amplitude, mV	150	10.7	5.7	4.0	20	4722	8.2	3.0	4.6	24
Conduction velocity, m/s	150	48.1	5.6	40.7	24	4659	44.7	4.8	38.9	14
F-min, ms	148	45.9	5.1	55.5	14	4686	48.0	5.4	54.9	16

**Peroneal nerve**										
Amplitude, mV	149	5.8	2.1	2.2	23	4824	4.4	1.8	2.6	28
Conduction velocity, m/s	150	47.1	5.0	40.5	24	4549	44.6	4.9	38.6	16
F-min, ms	145	44.6	5.9	53.2	17	4360	46.1	4.8	52.3	20

**Sensory nerves**										
**Sural nerve (orthodromic)**										
Amplitude, µV	150	8.9	7.5	1.3	6	3925	5.4	2.5	2.9	20
Conduction velocity, m/s	150	52.0	8.1	42.7	18	3916	49.2	6.9	40.2	10

**Superficial peroneal nerve**										
Amplitude, µV	129	6.8	4.5	1.5	6	1024	5.1	2.6	2.4	12
Conduction velocity, m/s	129	52.0	6.2	40.4	18	1013	45.5	5.2	38.6	9

**Medial plantar nerve**										
Amplitude, µV	129	3.7	4.8	0.7	14	1639	2.3	1.9	0.8	19
Conduction velocity, m/s	129	54.4	9.1	40.9	10	1638	50.0	7.7	40.9	9

IQR, inter-quartile range.

^a^Limit = Mean ±2 SD on transformed data for non-Gaussian distributions, retransformed to original scale.

^b^What percentage of NCS readings in the historical laboratory population would be classified as abnormal by the method.

When calculating e-norms limits by mean ±2.5 SD, the average percentage agreement increased marginally to 96 %, while the average abnormality ratios for children were reduced to 0.94 for children (range 0.6–2.2) and 0.77 for adults (range 0.3–2.5), i.e. a minor overcorrection. The biggest differences between individual reference limits were the same as for e-norms ±2 SD. Tables for mean ±2.5 SD can be found in the Appendix.

## 4 Discussion

We demonstrated that compared with traditionally derived reference limits, the standard e-norms method (±2 SD) produced slightly stricter reference limits on average, while increasing the range to ±2.5 SD resulted in more lenient reference limits. The e-norms method was streamlined by new software, and was further developed with the goal of increasing reliability in marker placings and to reduce rater confirmation bias.

The reference limits obtained through the standard e-norms method were slightly stricter on average (i.e., higher amplitudes and conduction velocities, lower F-waves). In essence, the e-norms method cuts off both tails of the historical laboratory distribution (cf. Fig. 2); This truncated distribution will then inevitably have low variance. Thus, by calculating reference limits based on this distribution with the norm of mean ±2 SD, it is likely the results would favor sensitivity over specificity. This could then help explain our findings of 15–30 % more abnormal NCS readings by e-norms limits on average. Our attempt at decreasing supposed false positives by calculating mean ±2.5 SD, instead lead to a minor overcorrection where e-norms classified 6–23 % fewer readings as abnormal.

The aim of this study was not to produce new reference limits, but to validate the [adapted] e-norms method. Still, marked differences between the e-norms method and traditional methods could impact diagnostics, for example our reported sensory nerve amplitudes. There are many possible explanations for why some larger differences occur, and a combination is likely. First, in general, performing standard mean ±SD calculations on non-Gaussian data distributions may lead to unintended results (e-norms plateaus are always truncated, often uniform). Since the purpose of such calculations is to exclude a certain percentage of tail-end cases (e.g. < 2.5 % and > 97.5 %), one may consider utilizing percentiles instead. This would annul the effect of non-Gaussian data distributions, and the decrease in precision due to interpolation is likely to be of little significance, especially in large samples. Our results support that such percentiles should be towards the extremes, e.g. top and bottom 1 %. This makes sense since the e-norms plateau per its definition already consists of a range where one is likely to find “normal values”, and narrowing the range further will only lead to stricter reference limits. Second, some e-norms plots were more difficult to rate than others. Although previous investigations have shown good inter-rater reliability (Earle and Jabre, 2020), our experience was that despite meeting before-hand, the raters had some difficulty applying the e-norms method consistently across all plots. This resulted in a few outlying reference limit that skewed the average results; For example, post-hoc review of non-blinded plots revealed that the large difference between medial plantar conduction velocity reference limits in children is likely attributable to faulty e-norms rating, and not necessarily the e-norms method per se. However, human raters are a necessary part of the method, and so our results should still be a realistic representation of applying e-norms on historical laboratory data. Third, poor stratification by age and height could play a role, i.e., pooling several subpopulations into one group. Although the groups of controls and age-matched historical data were relatively similar with regards to age and height, this may well change when the e-norms method is applied, as it excludes most supernormal or otherwise normal deviating values However, stratifying for narrower age groups and adjusting for height rapidly demands more data, which is an inherent challenge of the e-norms method, similar to the traditional method. Laboratories with smaller databases would still need to make well thought-out compromises regarding the most impactful variables, e.g. also stratify by height for conduction velocities or F-waves.

Marked differences in single NCS measures could also be attenuated by looking at the average nerve function of several relevant nerves when this is feasible (e.g., combined Z-scores of relevant NCS measures (Dunker et al., 2022)). In any case, when creating new e-norms derived reference limits (or any other) to be used in the clinic, it is advisable they be evaluated to ensure acceptable diagnostic face validity before implementing the results in clinical practice.

The e-norms reference limits are not being compared to an error-free “gold standard” per se, but the reference standard most commonly used. All reference limits are estimations with inherited uncertainties and assumptions (Haeckel et al., 2021), and because the statistical methods used in determining them cannot account for the overlapping NCS values between healthy and unhealthy patients, they are never perfect in discriminating between health and disease (Robinson, 2006). In the same vein, The mean ±2 SD paradigm of determining reference limits in a healthy sample is a pragmatic solution that’s deemed a decent trade-off between false positives and false negatives where neither demands priority, but is not a true illness-definition nor diagnostic end-all (Whyte and Kelly, 2018). Therefore, in the absence of a true “gold standard”, one can speculate whether the traditionally obtained reference limits or the e-norms limits leads to the best clinical decision, or if this lies somewhere in-between; In the end, what matters most is the intended purpose of the reference limits, i.e., whether it is more important to err on the side of reducing false positives or false negatives. Going forward, some nuance may be added by also comparing the e-norms derived reference limits to those obtained by other recently developed indirect methods, such as the E-Ref (Nandedkar et al., 2018), MeRef (Nandedkar et al., 2021) and mixture model clustering (MMC) (Reijntjes et al., 2021) methods. However, for the purpose of validation against the most established method, the e-norms method did produce somewhat stricter reference limits on average than those obtained through traditional means.

We made some adaptations to the e-norms method and compiled previous ideas into one easy-to-use software. In the e-norms method, the final decision of where to put the markers is subjective, although the use of a tangent and first-order derivatives gives the rater important cues. The idea of fitting a tangent to the plateau is simple, but often needs complementary information. For example, one can fit a tangent that gives the least possible residual sum of squares, or the longest possible tangent within a certain [subjective] tolerance limit of residual sum of squares. The use of derivatives helps this process, but it is not uncommon to e.g., encounter large spikes (single, large jumps in derivative values that are not part of a trend), especially if the e-norms dataset is small and/or parts of the expected distribution is poorly populated. By instead applying a moving average to the derivatives, these spikes are smoothed out, and the trend is much more easily identified. This should give the rater more reliable information on when a real change occurs. In addition to the moving average of the derivatives, adding a 3rd polynomial line has previously been done by Earle and Jabre (2020). This may be particularly useful in curves that appear jagged or display a more gradual change in slope toward the tails. Finally, similar to the work of Punga et al. (2019), our software can automatically hide the graph axes’ values. Obviously, this does not provide further technical aid in finding the most suitable inflection points, but reduces subconscious confirmation bias, though at the cost of easily interpreting NCS artefacts. Still, blinding the rater to the data assessed as the default option further substantiates the e-norms method as a data-neutral method with uses beyond clinical neurophysiology or clinical chemistry. Taken together, these adaptations provide the rater with a deeper toolbox which should serve to improve the reliability of the e-norms method, although future studies are necessary to determine the actual effects compared to the originally described e-norms method (Jabre et al., 2015).

The key strength of this study was that we applied the e-norms method to two large, real-life laboratory datasets, and compared the findings to sets of healthy controls collected by the same hospitals, with identical routines, protocols and training of personnel. In addition, confirmation bias towards known reference limits was reduced for all e-norms analyses by removing graph axes. To ensure that the e-norms method was applied appropriately and fairly for validation purposes, two blinded raters performed the analyses separately, and any disagreements were solved through discussion or a third blinded rater. An arguable weakness of the data analysis was that we chose not to perform data cleaning of the historical datasets, such as removal of outliers, artefacts or invalid measurements (e.g., conduction velocities or F-waves registered after motor amplitudes < 0.5 mV). However, the e-norms method is robust to outlying data, and thus the results should not be affected. In relation to this, we believe that broad adoption of methods such as e-norms is contingent on direct and automated use of raw data from the EMG machines, with the least possible input from the clinician or researcher.

Some key challenges still remain. First, the e-norms method is still dependent on some manual and subjective work from human raters, which, despite trained raters, may make it more inconsistent than fully automated methods. The subjective placement of e-norms markers also makes it impossible to calculate meaningful precision estimates of the final reference limits. Second, similar to other indirect methods of determining reference limits, the e-norms method is dependent on a relatively large amount of historical data for proper stratification (perhaps a couple hundred patients per age segment), which makes implementation difficult in new laboratories or when new equipment is adopted. This challenge grows when necessary to adjust for several independent variables, such as height, sex, BMI or temperature. For calculation of actual NCS reference limits by the e-norms method, two strategies are possible: 1) Limits must be stratified by each *important* covariate. Most NCS measures will depend statistically on at most two (or three) covariates (Falck et al., 1991), provided standardized limb-heating is applied as part of the NCS protocol (Stetson et al., 1992). Stratifying by these covariates may require a large number of NCS measurements not feasibly collected for some laboratories – possibly solved by a well standardized multi-center approach. 2) Extending the e-norms method by multivariate linear regression, like how the E-Ref method (Nandedkar et al., 2018) was extended by multivariate linear regression (Me-Ref) (Nandedkar et al., 2021). Future research should look into both the optimal approach to identify important covariates for each NCS measure, and the best methods for covariate adjustment. Third, the e-norms method could benefit from somehow including supernormal values (including short, lean subjects) and otherwise normal deviating values, e.g., height-related low conduction velocity and height or BMI related low sensory amplitudes. Lastly, large-scale NCS data is difficult to extract, compile and analyze: the data is often poorly standardized, stored in different, hard-to-read formats, and often kept in digital (or physical) silos for data protection and security purposes. Efforts are being made to standardize NCS data formats (Halford et al., 2021) and recording practices (Dunker et al., 2023). Meanwhile, data extraction is a hurdle for all data-driven methods that must be overcome locally until the industry catches up. Nonetheless, it is clear that as laboratory data does become more available, e-norms and other methods of analyzing historical data show great promise in developing ecologically valid reference limits, with the potential to improve neuromuscular diagnostics worldwide.

### 4.1 Conclusion

In conclusion, when compared to traditionally obtained reference limits for NCS in the lower limbs, the e-norms method (mean ±2 SD) yielded slightly stricter reference limits on average. However, some of this difference can be attributed to rater difficulties in applying the e-norms method correctly to certain plots. The largest discrepancies for individual NCS readings were found for tibial– and peroneal F-waves and sural– superficial peroneal and medial plantar sensory nerve readings. Calculating e-norms reference limits by mean ±2.5 SD led to an overcorrection and more lenient reference limits. To ensure high diagnostic validity of e-norms derived reference limits, future research should look into more accessible ways of adjusting for all relevant covariates. Our adapted e-norms software is user friendly and should further simplify the process of obtaining accurate and reliable reference limits for NCS.

## Funding

This work received financial support from the 10.13039/100010745Norwegian Medical Association’s Fund for Quality Improvement and Patient Safety, grant no. SAK2020002638.

## Author Contributions

ØD, HOPD, MUL and KBN conceptualized the study. ØD, MUL, PO, TS and KBN collected the data. ØD, TSS and JFJ further developed the methodology. ØD, HOPD, PO and TSS processed/analyzed the data. ØD and TSS created tables and figures. ØD wrote the final manuscript; all authors contributed to revision and editing of the final manuscript.

## Declaration of competing interest

The authors declare that they have no known competing financial interests or personal relationships that could have appeared to influence the work reported in this paper.
