# Supplementary material for: Harnessing historical data to derive reference limits – A comparison of e-norms to traditionally derived reference limits
Source: Clin Neurophysiol Pract. 2024 Apr 16;9:168–75. doi: 10.1016/j.cnp.2024.04.001 (PMC11067331; doi:10.1016/j.cnp.2024.04.001)
Supplement: Supplementary Data 1 [file mmc1.docx]

|  | **Traditional** | | | | | **E-norms** | | | | |
| --- | --- | --- | --- | --- | --- | --- | --- | --- | --- | --- |
| **Children 9-18 years** | n | Median | IQR | Reference limit (±2SD)^a^ | % abnormal in mixed dataset (±2SD)^b^ | n | Median | IQR | Reference limit (±2.5SD)^a^ | % abnormal in mixed dataset (±2.5SD)^b^ |
| **Motor nerves** |  |  |  |  |  |  |  |  |  |  |
| **Tibial nerve** |  |  |  |  |  |  |  |  |  |  |
| Amplitude, mV | 63 | 13.5 | 5.2 | 7.5 | 27 | 1658 | 10.7 | 3.6 | 5.5 | 20 |
| Conduction velocity, m/s | 62 | 48.4 | 5.3 | 41.0 | 11 | 1419 | 48.1 | 4.2 | 41.8 | 13 |
| F-min, ms | 63 | 39.4 | 7.4 | 51.8 | 9 | 1485 | 42.7 | 5.7 | 50.3 | 10 |
| **Peroneal nerve** |  |  |  |  |  |  |  |  |  |  |
| Amplitude, mV | 63 | 4.4 | 2.0 | 2.4 | 22 | 1683 | 4.1 | 1.5 | 1.7 | 15 |
| Conduction velocity, m/s | 63 | 50.9 | 4.8 | 43.2 | 16 | 1414 | 48.8 | 4.1 | 42.1 | 12 |
| F-min, ms | 62 | 40.0 | 7.4 | 50.1 | 10 | 1321 | 42.1 | 5.7 | 49.1 | 12 |
| **Sensory nerves** |  |  |  |  |  |  |  |  |  |  |
| **Sural nerve** |  |  |  |  |  |  |  |  |  |  |
| Amplitude, µV | 63 | 15.4 | 7.9 | 6.6 | 16 | 1301 | 14.3 | 6.5 | 6.0 | 13 |
| Conduction velocity, m/s | 63 | 56.0 | 8.5 | 44.7 | 9 | 1271 | 54.2 | 6.5 | 44.3 | 8 |
| **Superficial peroneal nerve** |  |  |  |  |  |  |  |  |  |  |
| Amplitude, µV | 63 | 8.4 | 5.1 | 4.0 | 12 | 687 | 8.4 | 3.8 | 3.6 | 10 |
| Conduction velocity, m/s | 63 | 53.4 | 6.8 | 44.3 | 11 | 1029 | 50.9 | 5.6 | 42.2 | 6 |
| **Medial plantar nerve** |  |  |  |  |  |  |  |  |  |  |
| Amplitude, µV | 63 | 10.1 | 5.9 | 3.8 | 13 | 228 | 8.4 | 3.7 | 3.5 | 12 |
| Conduction velocity, m/s | 63 | 59.6 | 9.2 | 45.3 | 6 | 222 | 59.6 | 6.4 | 48.9 | 13 |
|  |  |  |  |  |  |  |  |  |  |  |
| IQR, inter-quartile range ^a^Limit = Mean ± 2 SD / 2.5 SD on transformed data for non-Gaussian distributions, retransformed to original scale ^b^What percentage of NCS readings in the historical laboratory population would be classified as abnormal by the method | | | | | | | | | | |

Appendix 1: Reference limit and % abnormal in mixed dataset for ±2.5SD in children

Appendix 2: Reference limit and % abnormal in mixed dataset for ±2.5SD in adults

|  | **Traditional** | | | | | **E-norms** | | | | |
| --- | --- | --- | --- | --- | --- | --- | --- | --- | --- | --- |
| **Adults 20-44 years** | n | Median | IQR | Reference limit (±2SD)^a^ | % abnormal in mixed dataset (±2SD)^b^ | n | Median | IQR | Reference limit (±2.5SD)^a^ | % abnormal in mixed dataset (±2.5SD)^b^ |
| **Motor nerves** |  |  |  |  |  |  |  |  |  |  |
| **Tibial nerve** |  |  |  |  |  |  |  |  |  |  |
| Amplitude, mV | 144 | 12.6 | 5.6 | 5.8 | 11 | 3169 | 10.6 | 4.0 | 4.8 | 9 |
| Conduction velocity, m/s | 142 | 50.0 | 4.4 | 43.2 | 18 | 3121 | 48.2 | 4.5 | 42.6 | 16 |
| F-min, ms | 144 | 43.8 | 3.8 | 51.2 | 13 | 3223 | 44.6 | 4.0 | 51.0 | 13 |
| **Peroneal nerve** |  |  |  |  |  |  |  |  |  |  |
| Amplitude, mV | 146 | 5.8 | 2.5 | 2.8 | 16 | 3272 | 5.1 | 1.8 | 2.4 | 13 |
| Conduction velocity, m/s | 145 | 48.5 | 3.3 | 43.1 | 19 | 3072 | 47.9 | 4.4 | 40.5 | 11 |
| F-min, ms | 142 | 42.5 | 4.5 | 50.8 | 10 | 3051 | 43.3 | 4.5 | 50.4 | 11 |
| **Sensory nerves** |  |  |  |  |  |  |  |  |  |  |
| **Sural nerve (orthodromic)** |  |  |  |  |  |  |  |  |  |  |
| Amplitude, µV | 146 | 11.7 | 8.8 | 2.6 | 6 | 2805 | 7.2 | 3.3 | 3.1 | 9 |
| Conduction velocity, m/s | 146 | 53.9 | 7.9 | 42.5 | 8 | 2798 | 51.9 | 7.5 | 40.6 | 5 |
| **Superficial peroneal nerve** |  |  |  |  |  |  |  |  |  |  |
| Amplitude, µV | 122 | 8.3 | 6.1 | 2.9 | 7 | 767 | 8 | 3.7 | 3.3 | 10 |
| Conduction velocity, m/s | 122 | 52.2 | 7.1 | 43.0 | 19 | 758 | 48.3 | 5.3 | 40.1 | 8 |
| **Medial plantar nerve** |  |  |  |  |  |  |  |  |  |  |
| Amplitude, µV | 122 | 8.8 | 7.5 | 1.2 | 10 | 1269 | 4.5 | 3.6 | 0.7 | 5 |
| Conduction velocity, m/s | 122 | 56.7 | 8.7 | 45.3 | 14 | 1268 | 54.2 | 5.8 | 44.7 | 12 |
| **Adults 45-60 years** |  |  |  |  |  |  |  |  |  |  |
| **Motor nerves** |  |  |  |  |  |  |  |  |  |  |
| **Tibial nerve** |  |  |  |  |  |  |  |  |  |  |
| Amplitude, mV | 150 | 10.7 | 5.7 | 4.0 | 20 | 4722 | 8.2 | 3.0 | 3.7 | 19 |
| Conduction velocity, m/s | 150 | 48.1 | 5.6 | 40.7 | 24 | 4659 | 44.7 | 4.8 | 37.4 | 10 |
| F-min, ms | 148 | 45.9 | 5.1 | 55.5 | 14 | 4686 | 48 | 5.4 | 56.6 | 11 |
| **Peroneal nerve** |  |  |  |  |  |  |  |  |  |  |
| Amplitude, mV | 149 | 5.8 | 2.1 | 2.2 | 23 | 4824 | 4.4 | 1.8 | 2.2 | 24 |
| Conduction velocity, m/s | 150 | 47.1 | 5.0 | 40.5 | 24 | 4549 | 44.6 | 4.9 | 37.1 | 11 |
| F-min, ms | 145 | 44.6 | 5.9 | 53.2 | 17 | 4360 | 46.1 | 4.8 | 53.8 | 15 |
| **Sensory nerves** |  |  |  |  |  |  |  |  |  |  |
| **Sural nerve (orthodromic)** |  |  |  |  |  |  |  |  |  |  |
| Amplitude, µV | 150 | 8.9 | 7.5 | 1.3 | 6 | 3925 | 5.4 | 2.5 | 2.4 | 16 |
| Conduction velocity, m/s | 150 | 52.0 | 8.1 | 42.7 | 18 | 3916 | 49.2 | 6.9 | 37.9 | 5 |
| **Superficial peroneal nerve** |  |  |  |  |  |  |  |  |  |  |
| Amplitude, µV | 129 | 6.8 | 4.5 | 1.5 | 6 | 1024 | 5.1 | 2.6 | 1.9 | 9 |
| Conduction velocity, m/s | 129 | 52.0 | 6.2 | 40.4 | 18 | 1013 | 45.5 | 5.2 | 37.0 | 6 |
| **Medial plantar nerve** |  |  |  |  |  |  |  |  |  |  |
| Amplitude, µV | 129 | 3.7 | 4.8 | 0.7 | 14 | 1639 | 2.3 | 1.9 | 0.7 | 14 |
| Conduction velocity, m/s | 129 | 54.4 | 9.1 | 40.9 | 10 | 1638 | 50 | 7.7 | 38.4 | 5 |
|  | | | | | | | | |  |  |
| IQR, inter-quartile range ^a^Limit = Mean ± 2 SD / 2.5 SD on transformed data for non-Gaussian distributions, retransformed to original scale ^b^What percentage of NCS readings in the historical laboratory population would be classified as abnormal by the method | | | | | | | | | | |
